# Editorial: Induced pluripotent stem cells (iPSCs) for skeletal muscle diseases

**DOI:** 10.3389/fcell.2025.1556403

**Published:** 2025-01-29

**Authors:** Hidetoshi Sakurai, Masatoshi Suzuki, Atsushi Asakura

**Affiliations:** ^1^ Department of Clinical Application, Center for iPS Cell Research and Application (CiRA), Kyoto University, Kyoto, Japan; ^2^ Department of Comparative Biosciences, Stem Cell and Regenerative Medicine Center, University of Wisconsin-Madison, Madison, WI, United States; ^3^ Department of Biomedical Engineering, Stem Cell and Regenerative Medicine Center, University of Wisconsin-Madison, Madison, WI, United States; ^4^ Greg Marzolf Jr. Muscular Dystrophy Center and Department of Neurology, Stem Cell Institute, University of Minnesota Medical School, Minneapolis, MN, United States

**Keywords:** skeletal muscle, muscle stem cell, muscular dystrophgies, aging, IPSC, neuromuscular diseases, amyotrophic lateral sclerosis (ALS), satellite cell

Skeletal muscle is a highly regenerative tissue in the vertebrate body. This skeletal muscle regenerative capacity is mediated by muscle satellite cells (MuSCs), a stem cell population within skeletal muscle (Seale et al., 2001; Asakura et al., 2002). While MuSCs display some non-muscle differentiation potential, such as adipocytes, osteocytes, and fibroblasts *in vitro* or in some diseased environments (Asakura et al., 2001), their primary differentiation fate in normal muscle regeneration is skeletal muscle cells. MuSCs usually display a mitotically quiescent state and reside on the myofibers. However, during muscle regeneration, MuSCs are activated, exit the quiescent state, and give rise to myogenic precursor cells. After several rounds of cell proliferation, these myogenic precursor cells exit the cell cycle and fuse with each other to contribute to the newly formed myofibers. MuSC-derived myogenic precursor cells or myoblasts expanded *ex vivo* can be utilized for myoblast-mediated cell therapy for skeletal muscle diseases, including Duchenne muscular dystrophy (DMD) when injected into damaged myofibers via integration into myofibers (Peault et al., 2007; Perie et al., 2014; Skuk and Tremblay, 2015; Negroni et al., 2016). However, there are significant limitations to treatment in humans. The number of MuSCs and myoblasts available from human biopsies is limited. In addition, low cell viability and low contribution of transplanted cells have hindered their practical use in patients (Peault et al., 2007; Skuk and Tremblay, 2015; Negroni et al., 2016).

Human induced pluripotent stem cells (hiPSCs) are a type of pluripotent stem cells originally derived from adult somatic cells. These cells are genetically initialized to an embryonic stem cell (ESC)-like pluripotent state by expressing genes and factors that are essential for maintaining the characteristic properties of pluripotent stem cells. hiPSCs can be generated from human somatic cells from various tissues (Takahashi and Yamanaka, 2006; Takahashi et al., 2007; Chan et al., 2018). They are capable of self-renewal and can give rise to any cell type, including skeletal muscle cells and MuSCs, both *in vitro* and *in vivo*. Therefore, hiPSC-derived myogenic cells are a source of myogenic precursor cells that repair injured or diseased or aged skeletal muscle in patients after transplantation (Caron et al., 2023). In addition, the ability to capture the genetic diversity of different inherent muscle diseases and age-related muscle atrophy in an accessible culture system makes hiPSCs an attractive model source for generating myogenic cells for drug screening (Caron et al., 2023).

This Research Topic aims to highlight the progress of research on disease modeling using iPSCs and iPSC-based cell therapy in the field of skeletal muscle diseases. In particular, collecting technical tips for successful induction protocols of iPSC-derived skeletal muscle (Chal et al., 2016; Kodaka et al., 2017; Zhao et al., 2024) suitable for regenerative medicine and *in vitro* iPSC-based disease modeling is critical for understanding the molecular and cellular mechanisms of disease pathogenesis as well as drug screening.

Five primary research or review manuscripts were published on this Research Topic. In the first manuscript, Hamer and Rossi summarized the protocols to improve the generation, purification, and maturation of iPSC-derived myogenic cells for novel therapeutic applications. Fibro/adipogenic progenitors (FAPs) residing in skeletal muscle play essential roles in skeletal muscle homeostasis and regeneration via secreted molecules and remodeling of extracellular matrix (Molina et al., 2021). In diseased environments, FAPs give rise to adipogenic, osteogenic, or fibroblastic cells that significantly modulate normal muscle function. The second manuscript, by Zhao and Ikeya, summarized the current progress of the developmental origins and functions of FAPs as stromal cells of the muscle connective tissue (MCT) and the FAP-mediated heterotopic ossification causing the rare genetic disorder fibrodysplasia ossificans progressive (FOP) (Nakajima and Ikeya, 2019). In addition, this manuscript provides novel insights into the heterotopic ossification from patient-derived iPSCs. The third manuscript, by Xie et al., uncovered the molecular mechanism by which leukemia inhibitory factor (LIF) promotes the self-renewal and proliferation of MuSCs. Treatment with LIF also enhances the engraftment capability of MuSCs following injection into the skeletal muscle of DMD model *mdx* mice, suggesting the beneficial effect of LIF for hiPSC-derived MuSC transplantation therapy. The mutations of superoxide dismutase 1 (SOD1) gene have been known as a genetic cause of familial amyotrophic lateral sclerosis (ALS), a progressive neuromuscular disease without cure (Mead et al., 2023). In the fourth manuscript, Couturier et al. reported that hiPSC-derived myogenic cells carrying the *SOD1* D90A mutation display a reduction of several myogenic markers, including modulation of nuctic acetylcholine receptor (nAChR) cluster expression and the function, causing the morphological alteration of sarcomeres in myotubes. Therefore, the authors introduced the novel hiPSC-derived neuromuscular model for ALS (Lynch et al., 2019; Zhou et al., 2023). As humans age, changes in the composition of blood circulating factors are thought to contribute to the decline in muscle mass and strength. In the fifth manuscript, Tey et al. reported that supplementation with serum from aged rats significantly reduced cell proliferation, myotube formation, and myofiber maturation, significantly increased cell death, and induced transcriptomic changes in both ESC- and iPSC-derived myogenic cells, providing therapeutic applications to prevent age-related muscle weakness.

This series of five articles highlights the high quality of manuscripts published in the Research Topic of the Stem Cell Research section of Frontiers in Cell and Developmental Biology and reveals a research field that is moving forward at a remarkable pace.
